# Abnormal patellar loading may lead to femoral trochlear dysplasia: an experimental study of patellar hypermobility and patellar dislocation in growing rats

**DOI:** 10.1186/s13018-023-03500-6

**Published:** 2023-01-16

**Authors:** Shiyu Tang, Weifeng Li, Shengjie Wang, Fei Wang

**Affiliations:** Department of Joint Surgery, The Third Hospital of Hebei Medical, University, 139 Ziqiang Road, Shijiazhuang, 050051 Hebei China

**Keywords:** Femoral trochlear dysplasia, Patellar hypermobility, Patellar dislocation, Loading, Rat

## Abstract

**Background:**

This animal study aimed to explore the effects of patellar hypermobility and patellar dislocation on the developing femoral trochlea.

**Methods:**

Seventy-two 3-week-old Wistar rats were randomly divided into three groups. The sham group (SG) underwent simple incision and suture of the skin and subcutaneous tissue; the patellar hypermobility group (PHG) underwent medial and lateral retinacular release and pie-crusting technique for the patellar ligament; the patellar dislocation group (PDG) underwent plication of the medial patellofemoral retinaculum. Twelve rats in each group were euthanized at 3 and 6 weeks postoperatively, respectively, and specimens were collected. The bony sulcus angle (BSA), cartilaginous sulcus angle (CSA), trochlear sulcus depth (TSD), and thickness of the cartilage on the lateral facet (CTL), medial facet (CTM), and center (CTC) of the trochlea were measured on hematoxylin and eosin-stained sections.

**Results:**

In the PHG and PDG, the femoral condyles became blunt, the trochlear groove became shallower, and cartilage became thicker compared with the SG. Compared with the SG, the PHG and PDG had significantly larger BSA and CSA values at 3 (*p* < 0.05) and 6 weeks (*p* < 0.005), and a significantly shallower TSD (*p* < 0.05). At 3 weeks, all cartilage thicknesses in the PHG and the CTC and CTM in the PDG were significantly thinner than in the SG (PHG vs. SG: *p* = 0.009 for CTL, *p* < 0.001 for CTM, *p* = 0.003 for CTC; PDG vs. SG: *p* = 0.028 for CTC, *p* = 0.048 for CTM). At 6 weeks, the CTC was thicker in the PHG and PDG than the SG (PHG vs. SG: *p* = 0.044; PDG vs. SG: *p* = 0.027), and the CTL was thinner in the PDG than the SG (*p* = 0.044).

**Conclusion:**

Patellar hypermobility and patellar dislocation may result in trochlear dysplasia that worsens with age. Excessive or insufficient loading leads to trochlear dysplasia.

## Introduction

The patellofemoral joint is surrounded by multiple soft tissue structures, such as the medial patellofemoral retinaculum, lateral patellofemoral retinaculum, and the quadriceps and patellar tendons, which maintain the static and dynamic stability of the patella [1]. Li et al. [2] established an animal model of patellar dislocation by plicating the medial patellofemoral retinaculum. Huri et al. [3] created a model of patellar instability by releasing the medial patellofemoral ligament, medial patellomeniscal ligament, medial retinaculum, and the medial patellotibial ligament and capsule.

Trochlear dysplasia is an anatomical abnormality of the shape and depth of the trochlear groove [4]. As a major risk factor for patellofemoral instability, trochlear dysplasia has also been evaluated as a prognostic factor for recurrent patellar dislocation [5]. In 1964, Brattström [6] observed that patients with an unstable patella have a malformed trochlear groove. Malghem and Maldague [7] later quantified the trochlear depth on lateral radiographs. Subsequently, Dejour et al. [8] classified trochlear dysplasia based on computed tomography in the lateral view into Dejour type A (concave trochlea), type B (flat trochlea), type C (convex trochlea), and type D (severe trochlear dysplasia).

Several studies have reported that loading between the patella and the femoral trochlear groove is the key factor in shaping the femoral trochlea during its growth period. Li et al. [2] reported that patellofemoral instability may give rise to trochlear dysplasia in growing rabbits. Kaymaz et al. [9] created a simulated patella alta model via patellar tendon Z-plasty lengthening resulting in a flattened femoral groove. Yang et al. [10] demonstrated that insufficient pressure caused trochlear dysplasia in patellectomy model in growing rats. However, no study has investigated the role of patellar hypermobility due to ligamentous causes by medial and lateral patellar retinacular release on the development of the femoral trochlea in growing rats.

Joint hypermobility is defined as a single or generalized joint with a greater range of motion than a normal joint [11] and may lead to shoulder instability, osteoarthritis, scoliosis, fibromyalgia, and other diseases [12]. Wynne-Davies et al. [13] first described the relationship between joint hypermobility and dysplasia and proposed that hereditary joint hypermobility is one of the main causes of acetabular dysplasia. We defined patellar hypermobility as a greater patellar range of motion than in the normal patellofemoral joint and performed the present study to investigate the effect of patellar hypermobility on the femoral trochlea.

The aims of the present experimental study were: (1) to create a rat model of patellar hypermobility by releasing the medial and lateral patellar retinacula and to explore the influence of patellar hypermobility on the development of the femoral trochlea in growing rats; (2) to compare the influence of patellar hypermobility and patellar dislocation on the development of the femoral trochlea.

## Materials and methods

### Study design

This study was approved by the Medical Ethical Committee of the Hebei Medical University Third Hospital. Seventy-two 3-week-old female Wistar rats (weight 45–60 g) provided by Beijing Vital River Laboratory Animal Technology Co. Ltd. were used in this study. The animals were randomly divided into three groups. The sham group (SG, *n* = 24) underwent a sham surgical procedure, the patellar hypermobility group (PHG, *n* = 24) underwent medial and lateral patellar retinacular release and pie-crusting technique for the patellar ligament, and the patellar dislocation group (PDG, *n* = 24) received a destabilizing release of the medial retinaculum. Patellar dislocation merely means instability of patella in a lateral direction without real laxation. The assessment timepoints were 3 and 6 weeks after intervention. Twelve rats in each group were euthanized at each timepoint. The rats had free access to tap water and food and were kept in capacious plastic cages with a solid floor and adequate wood shavings in a calm and controlled environment with a 12-h light–dark cycle and a constant temperature of 25 ± 2 °C. All rats were euthanized by an overdose of pentobarbital sodium (200 mg/kg) in accordance with guidelines for animal euthanasia.

Surgery.

All surgical procedures were performed by the same senior surgeon. After an intraperitoneal injection of pentobarbital sodium (30 mg/kg), the rat was fixed to the operating table in the knee extension position. The operative area was shaved and sterilized using standard protocol.

**Fig. 1:**
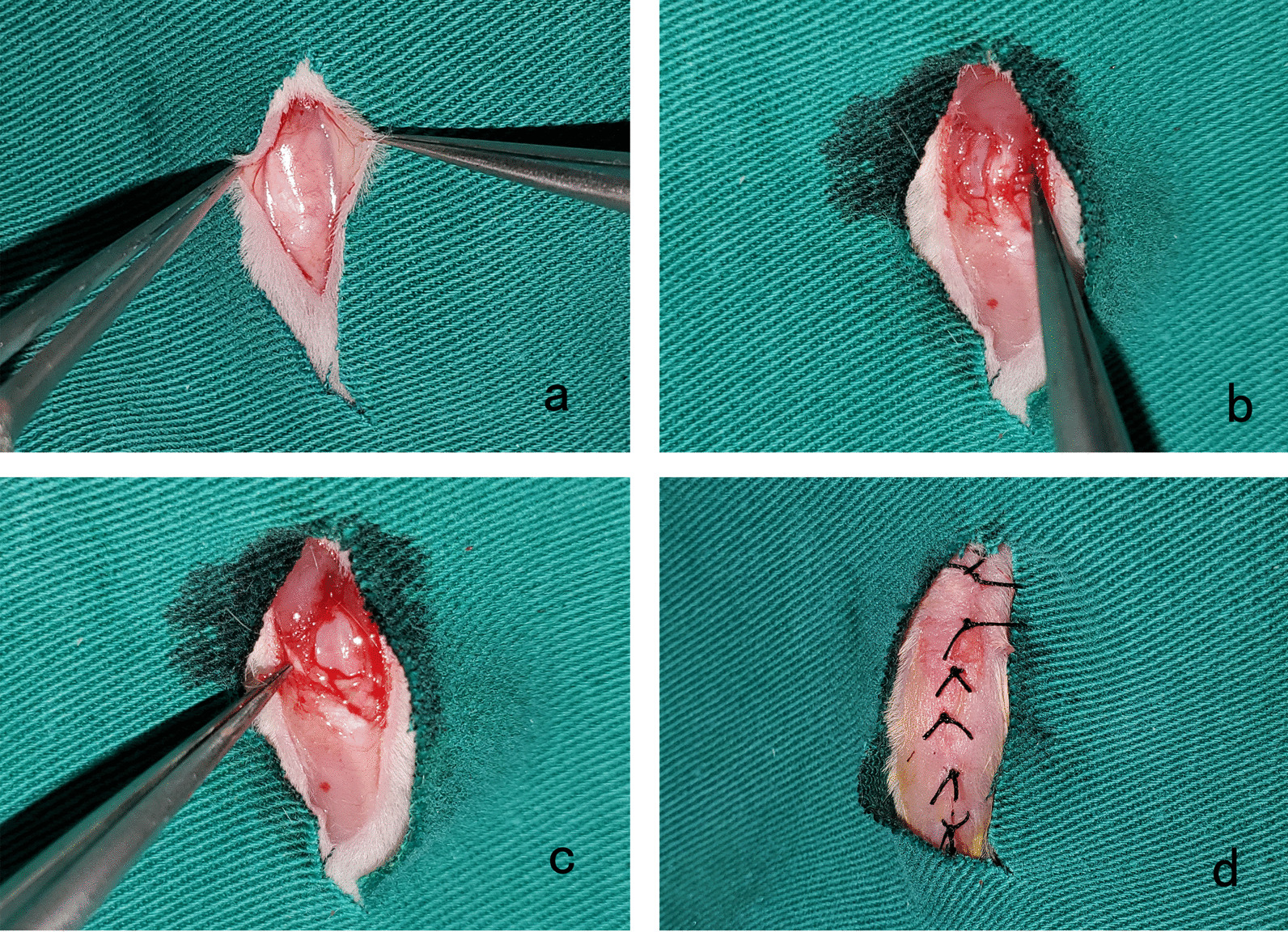
Detailed procedure of the patellar hypermobility group. **a**. Exposure of the joint capsule, **b.** incision of the medial retinaculum, **c**. incision of the lateral retinaculum, **d**. suturing of the incision after the operation

For the PHG, a midline skin incision was made on the right knee. The skin and subcutaneous tissue were separated until the patellar tendon and lateral and medial retinacula were exposed. Two incisions were made in the lateral and medial retinacula and the capsule along the edge of the patella, respectively. The patellar ligament received a pie-crusting technique using a No. 4.5 syringe needle, to increase patellar mobility. The patella was then pulled from the trochlear groove and restored to its normal position. A stable patellar trajectory without dislocation was observed during flexion and extension of the knee. After sufficient saline irrigation, the dissected skin and subcutaneous tissue were closed with interrupted sutures using 3–0 silk, without reconstruction of the medial and lateral patellofemoral retinacula and capsule (Fig. 1).

The SG underwent simple incision and suture of the skin and subcutaneous tissue of the right knee. The PDG had the medial patellofemoral retinaculum of the right knee plicated using the surgical method described in a previous study [2, 14].

### Postoperative care

Acetaminophen (30 mg/kg once daily) was administered as analgesia for 5 days postoperatively. Passive activity of the patella to the range of movement available during surgery and adequate exercise twice daily were initiated to prevent the formation of soft tissue adhesions and ankyloses. The animals were encouraged to move around in a big cage to ensure that they had adequate exercise.

### Histological analysis

**Fig. 2:**
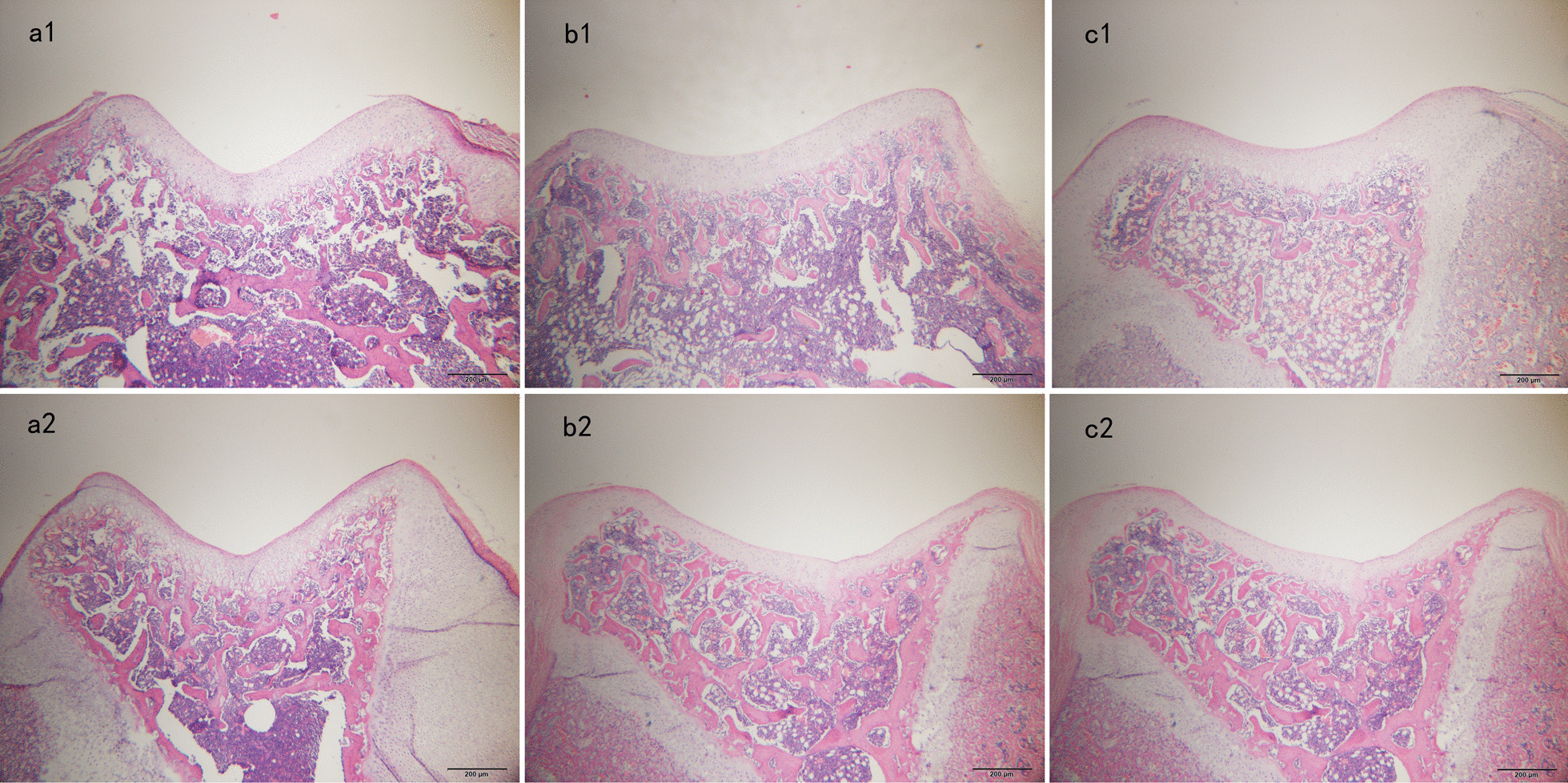
Histological examinations of axial sections of the femoral trochlea stained with hematoxylin and eosin. **a1** Sham group (SG) at 3 weeks postoperatively. **a2** SG at 6 weeks postoperatively. **b1** Patellar hypermobility group (PHG) at 3 weeks postoperatively. **b2** PHG at 6 weeks postoperatively. **c1** Patellar dislocation group (PDG) at 3 weeks postoperatively. **c2** PDG at 6 weeks postoperatively

Thirty-six rats (12 in the SG, PHG, and PDG, respectively) were euthanized at 3 and 6 weeks after surgery, respectively. Distal femoral tissue blocks were soaked in 10% neutral buffered formalin solution overnight at 4 °C. The tissue blocks were then rinsed in tap water for 2 h before being decalcified with 10% ethylenediaminetetraacetic acid for 30 days. The specimens underwent alcohol gradient dehydration and were embedded in paraffin. Then, 4 μm sections were cut perpendicular to the axial of the femoral shaft, and the sections were stained with hematoxylin and eosin. The prepared tissue sections were scanned with Olympus cellSens Entry 1.6 (Olympus Corporation, Tokyo, Japan) (Fig. 2).

**Fig. 3:**
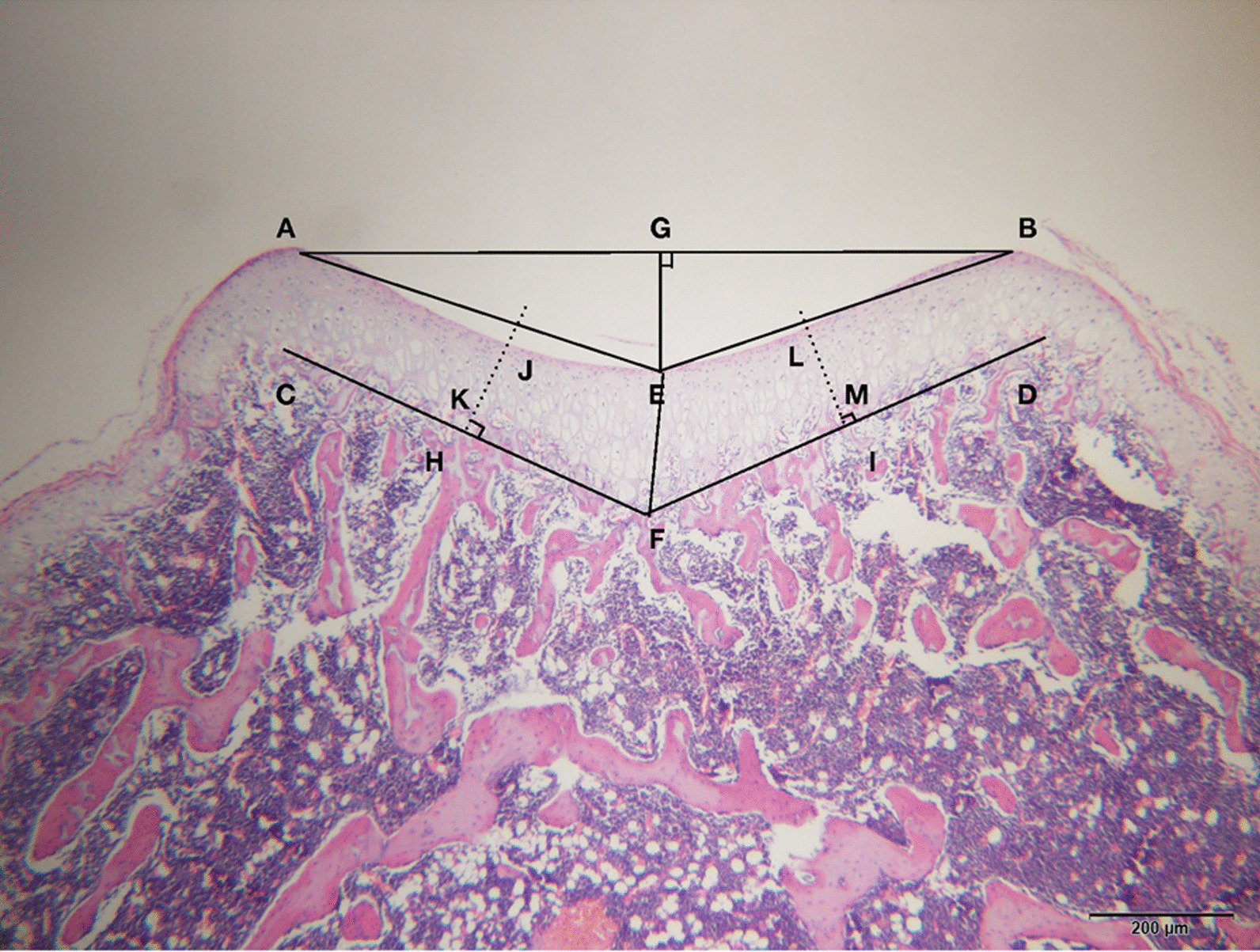
Tissue section of the trochlear groove. Points A and B are the highest points of the lateral and medial condyles of the femoral trochlea, respectively. E is the lowest point of the sulcus passing through point G, which is perpendicular to line A–B. Points C and D are the highest points of the bone of the lateral and medial condyles of the femoral trochlea. F is the deepest point of the bone of the trochlear groove. H and I are the midpoints of the lines C–F and F–D, respectively. The lines perpendicular to lines C–F and F–D at H and I, respectively, intersect with the cartilage of the trochlear sulcus and bone of the trochlear sulcus at points J, K, L, and M. Angle AEB is the cartilaginous sulcus angle, angle CFD is the bony sulcus angle, line E–D is the trochlear sulcus depth, the length of line J–K is the cartilage thickness at the lateral facet, the length of line E–F is the cartilage thickness at the center, and the length of line L–M is the cartilage thickness at the medial facet

The cartilaginous sulcus angle (CSA), bony sulcus angle (BSA), and trochlear sulcus depth (TSD) were measured on the scanned images. The CSA and BSA were defined as the angles formed between the lowest point of the pulley on the surface of bone or cartilage, respectively, and the highest point of both condyles. The depth of the trochlear sulcus was defined as the distance from the line connecting the medial and lateral condyles of the femur to the lowest point of the trochlear groove. Cartilage thickness was divided into the lateral cartilage thickness at the lateral facet (CTL), central cartilage thickness at the center (CTC), and medial cartilage thickness at the medial facet (CTM). The CTC was defined as the thickness of the deepest articular cartilage groove, and the CTM and CTL were defined as the thickness at the midpoint of the medial or lateral articular surface, respectively (Fig. 3).

### Statistical analysis

All data were statistically analyzed by SPSS 26.0 software (IBM, Chicago, IL, USA). Two-sample two-tailed Student’s *t* tests were used to compare the BSA, CSA, TSD, and cartilage thicknesses of the femoral trochlea between the two experimental groups and the SG. The CSA and BSA at different timepoints within the three groups were also compared by two-sample two-tailed Student’s *t* tests. The level of significance was set at 0.05.

## Results

### Findings at 3 weeks after surgical intervention

**Fig. 4:**
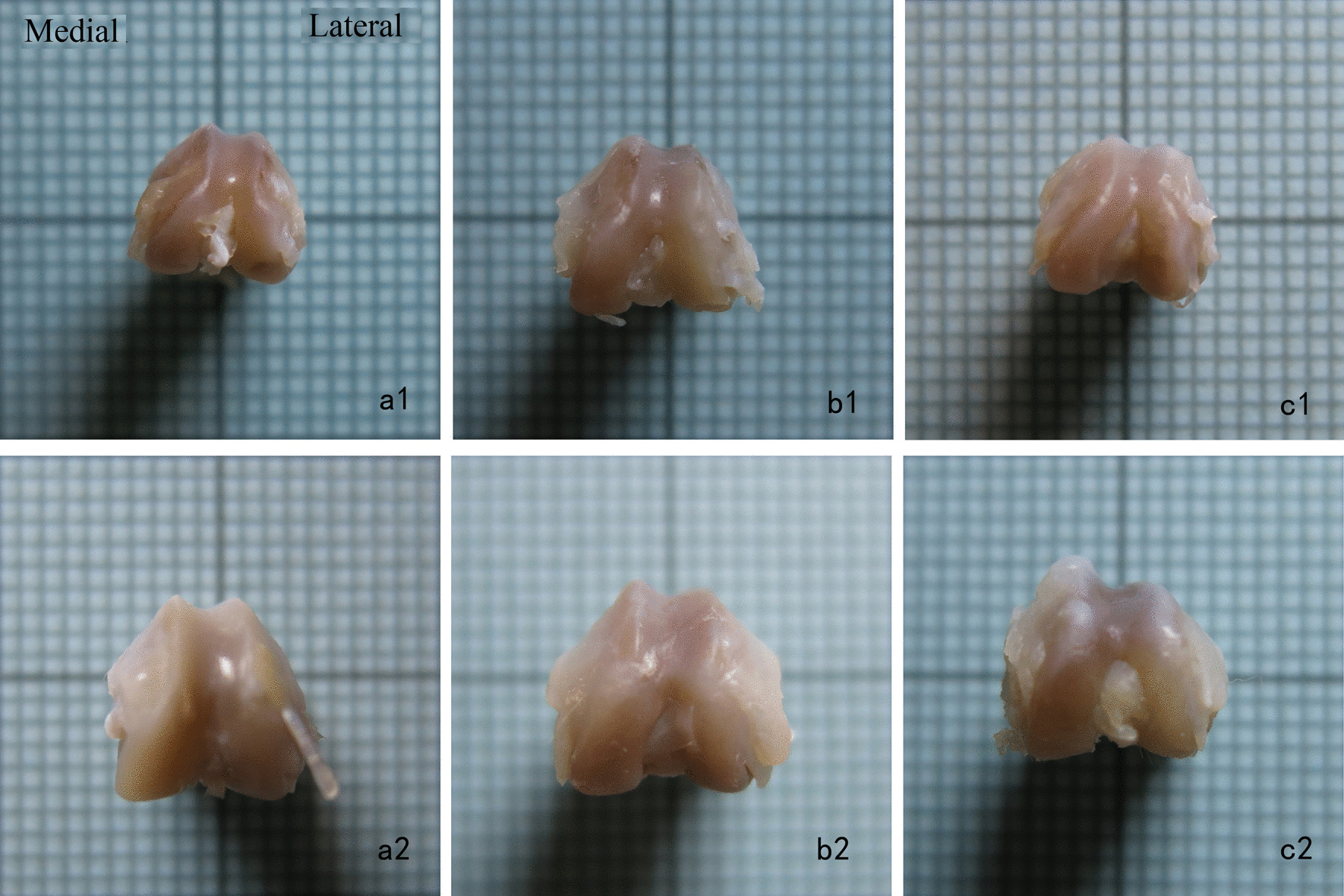
Gross anatomy of the femoral trochlea. **a1** Sham group (SG) at 3 weeks postoperatively. **a2** SG at 6 weeks postoperatively. **b1** Patellar hypermobility group (PHG) at 3 weeks postoperatively. **b2** PHG at 6 weeks postoperatively. **c1** Patellar dislocation group (PDG) at 3 weeks postoperatively. **c2** PDG at 6 weeks postoperatively. At 3 weeks postoperatively, the femoral trochlea was blunter in the PHG and PDG compared with the SG. At 6 weeks postoperatively, the trochlea groove was shallower in the PHG and PDG than the SG. At 6 weeks postoperatively, there was cartilage accumulation on the bilateral condyles in the PHG, and on the lateral condyle of the PDG

**Fig. 5:**
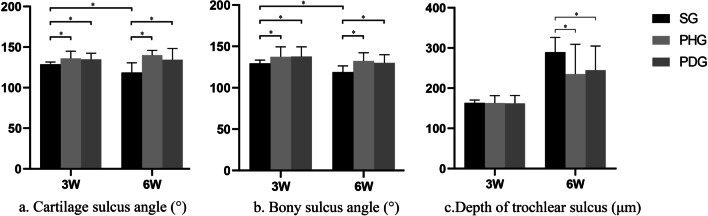
Angle and depth of trochlear sulcus at different timepoints. **a** The cartilaginous sulcus angle (CSA) was significantly larger in the patellar hypermobility group (PHG) and patellar dislocation group (PDG) than the sham group (SG) at both 3 and 6 weeks after surgical intervention. The CSA in the SG was significantly smaller at 6 weeks postoperatively than at 3 weeks postoperatively. **b** The bony sulcus angle (BSA) was significantly larger in the PHG and PDG than the SG at both 3 and 6 weeks after surgical intervention. The BSA in the SG was significantly smaller at 6 weeks postoperatively than at 3 weeks postoperatively. **c** At 6 weeks postoperatively, the depth of the trochlear sulcus (TSD) was significantly shallower in the experimental groups than the SG. Asterisks indicate significant differences. Error bars represent the 95% confidence intervals

**Fig. 6:**
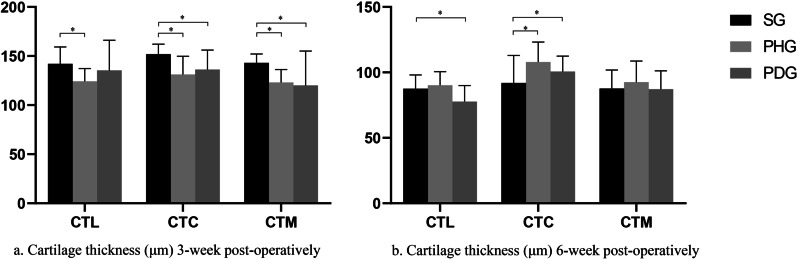
Cartilage thickness of trochlear sulcus at different timepoints. **a** Three weeks post-operation, all the cartilage thickness in the patellar hypermobility group (PHG) were significantly thinner when compared to the sham group (SG). The cartilage thickness at the medial facet (CTM) and cartilage thickness at the lateral facet (CTC) in the patellar dislocation group (PDG) were significantly thinner when compared to the SG. **b** Six weeks post-operation, the CTC was thicker in the PHG and PDG compared with SG. The CTL was thinner in the PDG in compared with SG, but did not show significant difference between PHG and SG, and the CTM in PHG and PDG was not statistically significant when compared to that in the SG. Asterisks indicate significant differences. Error bars represent the 95% confidence intervals

At 3 weeks after the surgical intervention, the femoral condyles in the PHG and PDG became blunt compared with those in the SG (Fig. 4). Compared with the SG, the PHG and PDG had a significantly larger BSA (PHG vs. SG: *p* = 0.045, PDG vs. SG: *p* = 0.036), larger CSA (PHG vs. SG: *p* = 0.018, PDG vs. SG: *p* = 0.008), and shallower TSD (PHG vs. SG: *p* = 0.038, PDG vs. SG: *p* = 0.037) (Fig. 5 and Table 1). All cartilage thicknesses were significantly thinner in the PHG than the SG (*p* = 0.009 for the CTL; *p* < 0.001 for the CTM; *p* = 0.003 for the CTC). Compared with the SG, the PDG had a significantly thinner CTM (p = 0.048) and CTC (*p* = 0.028), but a similar CTL (*p* = 0.519) (Fig. 6a and Table 1).

**Table 1:** Comparisons of the sulcus angle, sulcus depth, and cartilage thickness between groups

	PHG	SG	*t* value	*p*^a^	PDG	SG	*t* value	*p*^a^
*3 Weeks post-op*								
BSA°	137.5 ± 12.0	129.4 ± 4.1	−2.213*	**0.045**	137.7 ± 11.7	129.4 ± 4.1	−2.320*	**0.036**
CSA°	136 ± 9.0	128.7 ± 2.9	−2.693*	**0.018**	136.4 ± 8.1	128.7 ± 2.9	−3.116*	**0.008**
TSDμm	163.1 ± 18.3	176.1 ± 7.0	2.293*	**0.038**	162.2 ± 19.6	176.1 ± 7.0	2.315*	**0.037**
CTLμm	124.2 ± 13	142.1 ± 17.1	2.884	**0.009**	135.4 ± 30.7	142.1 ± 17.1	0.658*	0.519
CTCμm	131.3 ± 18.5	151.9 ± 10.2	3.389*	**0.003**	136.3 ± 19.9	151.9 ± 10.2	2.418*	**0.028**
CTMμm	123 ± 13.2	143 ± 9.2	4.316*	** < 0.001**	120.1 ± 34.9	143 ± 9.2	2.197*	**0.048**
*6 Weeks post-op*								
BSA°	132.4 ± 9.8	118.9 ± 7.5	−3.789	**0.001**	130.1 ± 9.8	118.9 ± 7.5	−3.118	**0.005**
CSA°	140.1 ± 5.8	118.5 ± 12.1	−5.562*	** < 0.001**	137.5 ± 12.3	118.5 ± 12.1	−3.813	**0.001**
TSDμm	235.1 ± 74	289.4 ± 36.8	2.273*	**0.037**	244.7 ± 60	289.4 ± 36.8	2.197*	**0.041**
CTLμm	90.2 ± 10.4	87.6 ± 10.4	−0.623	0.539	77.7 ± 12.2	87.6 ± 10.4	2.134	**0.044**
CTCμm	107.9 ± 15.3	91.9 ± 21	−2.133	**0.044**	100.6 ± 11.8	91.9 ± 21	−1.252*	**0.027**
CTMμm	92.6 ± 16.1	87.6 ± 14.2	−0.800	0.432	87.2 ± 13.9	87.6 ± 14.2	0.073	0.943

Asterisks indicate that the *t* test for equal variances was not assumed

Significant differences are marked in bold

*BSA* bony sulcus angle; *CSA* cartilaginous sulcus angle; *TSD* trochlear sulcus depth; *CTL* cartilage thickness at the lateral facet; *CTC* cartilage thickness at the center; and *CTM* cartilage thickness at the medial facet

^a^Two-sample Student’s *t* test

### Findings at 6 weeks after surgical intervention

At 6 weeks after surgical intervention, the trochlear groove was shallower in the PDG and PHG than the SG. There was cartilage accumulation on the bilateral condyles of the PHG, and on the lateral condyle of the PDG (Fig. 4). Compared with the SG, the PHG and PDG had a significantly larger BSA (PHG vs. SG: *p* = 0.001, PDG vs. SG: *p* = 0.005) and CSA (PHG vs. SG: *p* < 0.001, PDG vs. SG: *p* = 0.001) (Fig. 5a, b and Table 1), and a significantly shallower TSD (PHG vs. SG: *p* = 0.037, PDG vs. SG: *p* = 0.04) (Fig. 5c and Table 1). The CTC was thicker in the PHG and PDG than the SG (PHG vs. SG: *p* = 0.044, PDG vs. SG: *p* = 0.027). The CTL was thinner in the PDG than the SG (*p* = 0.044), but did not significantly differ between the PHG and SG (*p* = 0.539). The CTM did not significantly differ between the PHG and PDG and the SG (PHG vs. SG: *p* = 0.432, PDG vs. SG: *p* = 0.943) (Fig. 6b and Table 1).

### Findings at 3 versus 6 weeks after surgical intervention

The SG had a significantly smaller BSA (*p* < 0.001) and CSA (*p* < 0.001) at 6 weeks after surgical intervention compared with 3 weeks after surgical intervention. In the PHG and PDG, there were no significant differences between timepoints in the CSA (PHG: *p* = 0.544, PDG: *p* = 0.257) or BSA (PHG: *p* = 0.272, PDG: *p* = 0.098) (Fig. 5a, b and Table 2).

**Table 2:** Comparison of the BSA and CSA between the two timepoints within each group

	3 Weeks post-op	6 Weeks post-op	*t* Value	*p*^a^
*BSA°*				
SG	129.4 ± 4.1	118.9 ± 7.5	4.216*	**0.001**
PHG	137.5 ± 12.0	132.4 ± 9.8	1.127	0.272
PDG	137.7 ± 11.7	130.1 ± 9.8	1.727	0.098
*CSA°*				
SG	128.7 ± 2.9	118.5 ± 12.1	2.825*	**0.015**
PHG	136 ± 9.0	140.1 ± 5.8	−1.325*	0.201
PDG	136.4 ± 8.1	137.5 ± 12.3	−0.249	0.806

Asterisks indicate that the *t* test for equal variances was not assumed

Significant differences are marked in bold

*BSA* bony sulcus angle; *CSA* cartilaginous sulcus angle; *SG* sham group; *PHG* patellar hypermobility group; and *PDG* patellar dislocation group

^a^Two-sample Student’s *t* test

## Discussion

Our novel animal model in which the medial and lateral retinacula of the patella were released including destabilizing the patellar ligament showed that patellar hypermobility influenced the development of the femoral trochlea and led to trochlear dysplasia, which worsened with age. Both patellar hypermobility and patellar dislocation influenced the development of the femoral trochlea, mainly expressed as abnormal trochlear shape and cartilage thickness. The loading on the femoral trochlea greatly affected the developing trochlea. Excessive or insufficient loading may cause thinning and deformation of the cartilage and bone.

The setting of two experimental groups (PHG and PDG) and a control group (SG) increased the reliability and sensitivity of the present study. Previous research has found that excessive mechanical loading above the lateral condyle of the femur following patellar dislocation may result in a shallower trochlear groove and lower lateral condyle [2]. However, patients with joint hypermobility have a high incidence of femoral trochlear dysplasia [15]. Ueda et al. [16] observed the ultrastructure of the skin and patellofemoral ligament in dogs by histology and electron microscopy and found that patella dislocation and trochlear dysplasia were associated with overextended skin. Patellar dislocation model may increase stresses on the lateral facet and decrease them on the medial facet [17].

The patellofemoral joint is attached to multiple soft tissue structures to maintain the stability of the patella and ensure the range of motion of the knee joint. The medial patellar retinaculum consists of the medial patellofemoral ligament, medial patellotibial ligament, and medial patellomeniscal ligament and provides the lateral stability of the patella [18], with the medial patellofemoral ligament accounting for 53%–67% of the medial restraining force [19]. Lateral instability due to an insufficient medial patellofemoral ligament has been extensively confirmed anatomically and biomechanically [20]. The lateral patellar retinaculum consists of the iliotibial band, lateral patellofemoral ligament, and lateral patellotibial ligament and provides the medial stability of the patella [21]. A cadaveric study confirmed that the lateral retinaculum restrains the lateral translation of the patella in an extended knee [22]. Furthermore, the lateral patellofemoral ligament is important in protecting the patella from medial instability [23]. In the present study, the release of the entire medial and lateral stability structures of the patella resulted in patellar hypermobility, loosening of the patellofemoral joint, and finally gave rise to obvious femoral trochlear dysplasia in growing rats. However, whether the release of either lateral or medial retinaculum may lead to hypoplasia of the lateral or medial femoral condyle, respectively, could be a subject of a future study involving experimental design with release of medial retinaculum only and lateral retinaculum only compared to a control group.

The changes in the geometrical morphology of the femoral trochlea over time remain controversial. Nietosvaara et al. [24] measured the CSA in 50 normal children on ultrasonography and reported no significant change in the angle with increasing age. Furthermore, a retrospective analysis of magnetic resonance images of adolescents with trochlear dysplasia reported no significant differences in the shape of the dysplastic trochlea [4]. In contrast, Øye et al. [25] used ultrasonography to track the femoral trochlear groove in 174 newborns until the age of 6 years and found significant differences between the normal group and the trochlear dysplasia group in the changes in the trochlear groove angle; the trochlear groove angle of the normal group increased, while the angle of the trochlear dysplasia group decreased. However, all of the above-mentioned studies measured the femoral trochlear groove as the CSA on ultrasonography or magnetic resonance images. The present study revealed that the BSA and CSA decreased significantly (by 8.1% for the BSA and by 7.9% for the CSA) with age in the normal biomechanical environment of the femoral trochlea in the SG. However, the PHG and PDG showed no significant changes in the CSA or BSA over time, which was probably due to the abnormal molding of cartilage and bone under abnormal loading, and only showed changes in the linear measurements of the femoral trochlea without changes in morphological variables such as the trochlear sulcus angle.

It remains unclear whether femoral trochlear dysplasia is congenital due to genetic factors or occurs due to insufficient loading. Glard et al. [26] reported that the CSA in the fetus appears to be the same as that in adults and is independent of age and sex. Similarly, Parikh et al. supported the genetic origin of trochlear dysplasia [4], and Miller et al. [27] declared that recurrent patellar dislocation appears to be inherited. In addition, Dejour et al. [28] reported that 96% of patients with a history of patellar dislocation have radiographic evidence of trochlear dysplasia. However, there is currently no direct evidence of the genetic origin of trochlear dysplasia. Numerous scholars believe that abnormalities in the static and dynamic relationships between the patella and femoral trochlea fail to appropriately stimulate the femoral trochlea, resulting in femoral trochlear dysplasia. The shape of developing bone is altered in response to function, and the architecture of cancellous bone changes with mechanical stress [29]. A case report of a 16-year-old boy with trochlear dysplasia after a below-knee amputation confirmed that certain biomechanical input is essential for the formation of the trochlear groove [30]. A linear relationship between trochlear dysplasia and the pressure in the patellofemoral joint has been demonstrated in animal models of patellar dislocation [2], patella alta [9], and patellar resection [31]. In the current study, the PHG and PDG developed trochlear dysplasia, which was expressed as a shallower and wider trochlear groove that worsened with age. The present findings support the theory that loading of the femoral trochlea is a key factor in the development of the trochlea, and that excessive or insufficient loading leads to trochlear dysplasia.

Articular cartilage is mainly composed of chondrocytes and extracellular matrix. The synthesis and metabolism of the extracellular matrix is regulated by chondrocytes, and the main pressure-bearing structural components of chondrocytes are collagen and proteoglycan. The thickness and geometry of cartilage are related to the loading of the joint during development [32]. In an embryonic chick model, immobilization decreases the cartilage matrix formation and mechanical properties of the tibiofemoral articular cartilage of fixed embryos compared with controls [33]. Hagiwara et al. [34] confirmed that decreasing the load also creates catabolic responses in the articular cartilage of rats with the knee fixed in flexion. In agreement with these previous studies, the present study showed that the full-layer cartilage thickness of the trochlear groove in the PHG and the medial and central cartilage thicknesses in the PDG were significantly thinner at 3 weeks postoperatively compared with the SG; compared with the SG, the PHG showed a 12.6% decrease in the CTL, 13.6% decrease in the CTM, and 14.0% decrease in the CTM, while the PDG showed a 10.3% in the CTM and 16.0% decrease in the CTM. These differences may be because reduced loading created catabolic responses. However, at 6 weeks after surgical intervention, the CTC was significantly thicker in the PHG and PDG than the SG (by 17.4% in the PHG and by 9.4% in the PDG), while the CTM was similar in the PHG, PDG, and SG. This may be due to the consistent matching morphology of the patella and trochlear groove following animal growth, resulting in a balance between cartilage catabolism and anabolism in accordance with the alterations in pressure on the cartilage. An in vitro bovine cartilage explant system showed that excessive mechanical stress damages the extracellular matrix, changes the balance of chondrocytes, and finally results in catabolism exceeding anabolism [35]. However, caution is needed when comparing the in vivo situation to the results of in vitro experiments. In the present in vivo experiment, the CTL of the PDG was significantly thinner (by 11.3%) at 6 weeks postoperatively, which agreed exceptionally well with the findings of the previous study. Of note, there was no significant difference between the PHG and SG in the TCL and TCM. Although there was a gradual matching of the physiological morphology with growth and development as stated above, the cartilage damage caused by overload has poor repairability, while the cartilage thinning caused by load reduction has good reversibility. In healthy knee joints, the articular cartilage is usually thicker in the center of the groove, and the evaluation of the femoral groove is more accurate when it is based on the shape of cartilage than bone [36]. Similar results were found in the present study. In addition, loading changes the bone shape by affecting osteoblasts and osteoclasts to add or remove bone to the appropriate surface [37]. Biomechanics play an important role in bone formation, which begins with a primary cartilaginous matrix that later calcifies and forms bone via the endochondral ossification process [38]. In the present study, the BSA and CSA in the SG were significantly smaller at 6 weeks after surgical intervention than at 3 weeks postoperatively. The alteration of the cartilage and bone of the trochlea with aging supports the previous findings.

Articular cartilage is a highly differentiated tissue that has limited regenerative capacity due to its avascularity [39]. Long-term patellar dislocation may lead to osteoarthritis [40]. Previous studies showed that trochlear dysplasia is improved by early reduction of patellar subluxation, which reduces the risk of secondary surgery and protects the articular cartilage [14, 41]. Rajdev et al. [42] held that trochlear remodeling is allowed for patients at a younger age (< 10 years). The present study simulated patellar hypermobility and patellar subluxation, both of which resulted in trochlear dysplasia. This suggests that patients with patellar hypermobility and even patients with global joint hypermobility may be at risk of developing trochlear dysplasia. If the present findings are confirmed in further studies, measurements of patellar hypermobility may become part of routine monitoring and prevention of trochlear dysplasia. In addition, in clinical operations performed to adjust the load between the patella and the femoral trochlea in adolescents, the tension of the reconstructed ligaments should be adjusted to avoid excessive or insufficient tension.

The present study has some limitations. First, the animal model does not completely match the biomechanics and anatomical structure of the human patellofemoral joint, which may lead to errors. Second, the cross-sectional design is inferior to longitudinal studies that can better assess growth over time; however, there are few methods for assessing cartilage in living animals. Third, the cartilage thickness may have been underestimated due to histological management, as the processes of fixation, decalcification, and staining may change the cartilage thickness. Fourth, there were only two assessment timepoints. Fifth, the dislocation group might not be as standardized as presented. In future, the number of sampling timepoints will be increased to closely observe the changes in the femoral trochlear bone and cartilage with age.

## Conclusion

Patellar hypermobility and patellar dislocation may result in femoral trochlear dysplasia that worsens with age. The loading on the femoral trochlea is a key factor in the development of the trochlea. Excessive or insufficient loading leads to trochlear dysplasia.

## Data Availability

The datasets used and/or analyzed during the current study are available from the corresponding author on reasonable request.

## Abbreviations

SG: Sham group
PHG: Patellar hypermobility group
PDG: Patellar hypermobility group
BSA: Bony sulcus angle
CSA: Cartilaginous sulcus angle
TSD: Depth of trochlear sulcus
CTL: Thickness of cartilage on the lateral facet
CTM: Thickness of cartilage on the medial facet
CTC: Thickness of cartilage on the central facet
