# Correction: Abnormal patellar loading may lead to femoral trochlear dysplasia: an experimental study of patellar hypermobility and patellar dislocation in growing rats

**DOI:** 10.1186/s13018-026-06839-8

**Published:** 2026-04-16

**Authors:** Shiyu Tang, Weifeng Li, Shengjie Wang, Fei Wang

**Affiliations:** Department of Joint Surgery, The Third Hospital of Hebei Medical, University, 139 Ziqiang Road, Shijiazhuang, 050051 Hebei China

**Correction to: Journal of Orthopaedic Surgery and Research (2023) 18:39** 10.1186/s13018-023-03500-6

In the original version of this article, an error occurred during the final assembly of the schematic figures. Panel b2 of Figure 2 was inadvertently published as a duplicate of panel c2. The correct and incorrect version of the figures are given below.

Incorrect Fig. 2

**Fig. 2:**
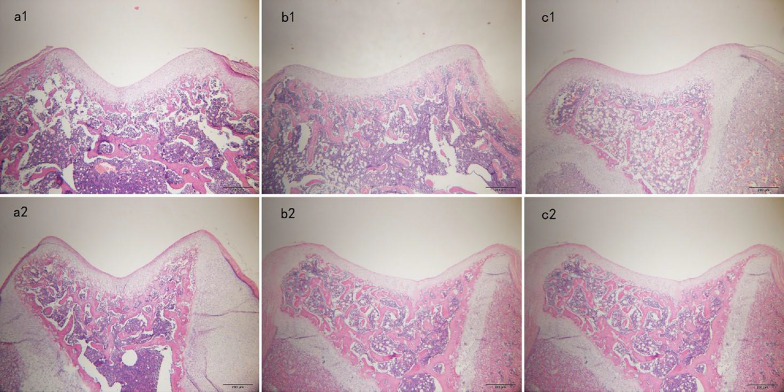
Histological examinations of axial sections of the femoral trochlea stained with hematoxylin and eosin. **a1** Sham group (SG) at 3 weeks postoperatively. **a2** SG at 6 weeks postoperatively. **b1** Patellar hypermobility group (PHG) at 3 weeks postoperatively. **b2** PHG at 6 weeks postoperatively. **c1** Patellar dislocation group (PDG) at 3 weeks postoperatively. **c2** PDG at 6 weeks postoperatively

Correct Fig. 2

**Fig. 2:**
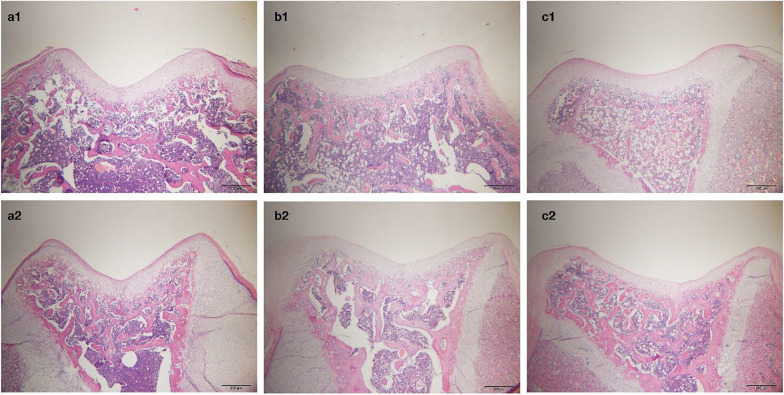
Histological examinations of axial sections of the femoral trochlea stained with hematoxylin and eosin. **a1** Sham group (SG) at 3 weeks postoperatively. **a2** SG at 6 weeks postoperatively. **b1** Patellar hypermobility group (PHG) at 3 weeks postoperatively. **b2** PHG at 6 weeks postoperatively. **c1** Patellar dislocation group (PDG) at 3 weeks postoperatively. **c2** PDG at 6 weeks postoperatively
